# Association between COVID-19 Diagnosis and Coronary Artery Thrombosis: A Narrative Review

**DOI:** 10.3390/biomedicines10030702

**Published:** 2022-03-18

**Authors:** Francesco Nappi, Omar Giacinto, Omar Ellouze, Antonio Nenna, Sanjeet Singh Avtaar Singh, Massimo Chello, Assine Bouzguenda, Xavier Copie

**Affiliations:** 1 Department of Cardiac Surgery, Centre Cardiologique du Nord de Saint-Denis (CCN), 36 Rue des Moulins Gémeaux, 93200 Saint-Denis, France; 2 Cardiovascular Surgery, Università Campus Bio-Medico di Roma, 00128 Rome, Italy; o.giacinto@unicampus.it (O.G.); a.nenna@unicampus.it (A.N.); m.chello@unicampus.it (M.C.); 3 Department of Anesthesia, Centre Cardiologique du Nord, 93200 Saint-Denis, France; ellouze.omar@yahoo.fr (O.E.); dr.hassine.bouzguenda@gmail.com (A.B.); 4 Department of Cardiothoracic Surgery, Aberdeen Royal Infirmary, Aberdeen AB25 2ZN, UK; sanjeetsinghtoor@gmail.com; 5 Department of Arrhythmology and Electrostimulation, Centre Cardiologique du Nord, 93200 Saint-Denis, France; x.copie@ccn.fr

**Keywords:** SARS-CoV-2 infection, COVID-19, coronary artery thrombosis, neutrophil extracellular traps (NETs)

## Abstract

Coronavirus disease 2019 is characterized by its severe respiratory effects. Data early on indicated an increased risk of mortality in patients with cardiovascular comorbidities. Early reports highlighted the multisystem inflammatory syndrome, cytokine storm, and thromboembolic events as part of the disease processes. The aim of this review is to assess the association between COVID-19 and its thrombotic complications, specifically related to the cardiovascular system. The role of neutrophil extracellular traps (NETs) is explored in the pathogenesis of the disease. The structure and anatomy of the virus are pivotal to its virulence in comparison to other α and β *Coronaviridae* (HCoV-229E, HCoV-OC43, HCoV-229E, HCoV-NL63, HCoV-OC43, and HCoV-HKU1). In particular, the host interaction and response may explain the variability of severity in patients. Angio tensin-converting enzyme 2 (ACE2) activation may be implicated in the cardiovascular and throm bogenic potential of the disease. The virus may also have direct effects on the endothelial lining affecting hemostasis and resulting in thrombosis through several mechanisms. Dipyridamole may have a therapeutic benefit in NET suppression. Therapeutic avenues should be concentrated on the different pathophysiological steps involving the virus and the host.

## 1. Introduction

Coronavirus disease-2019 (COVID-19) is a viral disease triggered by severe acute respiratory syndrome coronavirus 2 (SARS-CoV-2) [1]. Epidemiological data show a cardiac implication in COVID-19 infection, which is a major concern as it affects the population in different ways. The spread of SARS-CoV-2 worldwide has seen an exponential increase in the severity and mortality of the disease for the age group beyond the sixth decade of life, especially those with cardiovascular comorbidities [2].

During SARS-CoV-2 infection, patients with previous cardiovascular diseases may be at increased risk for COVID-19-linked adverse events. Likewise, several reports have suggested that, even in patients without a history of cardiovascular disease, there was a potential risk of developing cardiovascular complications [3,4,5].

Since the first spread of the infection in Wuhan, patients admitted to the hospital with COVID-19 have presented notable cardiac injuries, highlighting a substantial statistical convergence between cardiac damage and mortality during hospitalization. Patients who experienced cardiac injury were older with more comorbidities when compared to those without cardiac injury [6]. The resulting higher leukocyte counts prompted investigators to be weary of multisystem inflammatory syndrome, cytokine storm, and thromboembolic events as nefarious complications of COVID-19 [6,7,8,9,10,11,12,13,14].

We learned that patients with COVID-19 had a predisposition to thrombotic disease in both venous and arterial circulation, which potentially drives the development of thrombotic coronary obstruction and myocardial infarction [15,16]. Viral infiltration of myocardial cells provokes an inflammatory storm that may cause overexpression of cytokines with consequently higher prothrombotic status, which continuously spirals as a potentially developing thrombosis process. It has been suggested that organ dysfunction in severe SARS-CoV-2 infection is related to the formation of neutrophil extracellular traps (NETs) [17].

The process leading to NET generation is called NETosis. It is related to a particular type of cell death, diverging from the biological process of necrosis and apoptosis. Despite viruses being recognized for their propensity to circumvent the body’s immune response and trigger the NETosis processes, evidence has only recently emerged in the medical literature on the role of NETs in COVID-19 infection [18,19,20,21,22].

Evidence from autopsy studies suggests a vascular-obstructing process caused by aggregated NETs as a relevant pathogenic mechanism [23]. However, this pandemic has taught us that autopsies should not be delayed even in this setting, as a complete understanding of viral pathophysiology and organ damage is crucial for treatment and to reduce disease-related morbidities [24]. Simultaneously, during the pandemic, several reports have recorded poorer results relating to the treatment of STEMI (ST-elevation myocardial infarction) [25,26]. The results of these studies indicated that the prognosis was worse among patients with SARS-CoV-2 infection than among those without infection [27,28,29]. Investigators evaluated out-of-hospital or in-hospital STEMI in patients diagnosed with COVID-19, revealing a higher mortality rate than in patients without viral infection [30]. During the course of COVID-19 in patients with cardiac damage, myocardial enzyme levels were elevated, resulting in worsening cases of deteriorating clinical conditions [31].

We revisited and discussed the molecular aspect of the thrombophilic state in SARS-CoV-2 infection secondary to inflammation, platelet activation, endothelial dysfunction, and stasis. We paid particular attention to the mechanisms of coronary thrombosis. We believe that the data presented here could provide a basis for further judgment on the coronary artery thrombosis process during COVID-19 and could help the biologist–physician–patient discussion of risks and expectations after SARS-CoV-2 infection.

## 2. Structure and Genomics of SARS-CoV-2

The structure and genetics of SARS-CoV-2 are crucial to understand disease pathophysiology and to develop drugs or vaccines. These features are also relevant for their implementation and diffusion among people. SARS-CoV-2 has a spherical geometrical form with a single strand of positive-sense RNA [32,33,34] and relies on multiple components for cell adhesion and replication: different surface spike glycoproteins (S) which allow the adhesion to the cell membrane and induce antibody neutralization, an envelope protein membrane (E), structural membrane proteins such as M-protein (M), and the positive-sense single-stranded RNA with its structurally associated proteins such as nucleoprotein (N) [35,36,37,38,39,40,41,42].

SARS-CoV-2 has peculiar genomic variations compared to SARS-CoV and MERS-CoV, resulting in a more virulent virus. For instance, a single-nucleotide mutation in the S protein (N501T) enhances the viral binding to angiotensin-converting enzyme type 2 (ACE2) receptors of host cells [43].

Biologists and geneticists have worked to identify six strains of SARS-CoV-2 [44,45]. G and GR variants are more common in Europe, while GH is frequently found in North America (Table 1).

## 3. SARS-CoV-2 Host Interaction

### 3.1. Host Cell Interaction

**Figure 1:**
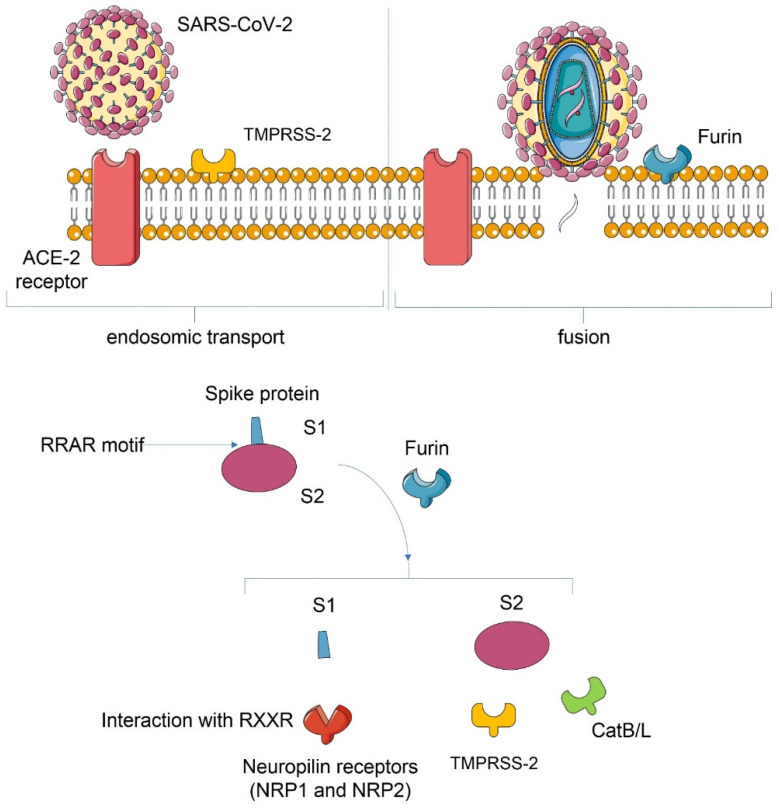
Host cell interaction. S protein has a polybasic sequence motif constituted by Arg–Arg–Ala–Arg (RRAR) in S1/S2 boundary site. This is a cleavage point for the protein convertase furin [48,49]. After furin interaction, S1 and S2 are noncovalently linked with Transmembrane serine protease 2 (TMPRSS2), further priming S2. This process has a key role in the enhanced spreading of SARS-CoV-2, matching it with other viruses with the same site of anchorage upon the cell surface. Polybasic cleavage site causes a more pathogenic action of virus, enabling it to fuse with the host cell. Furin cleavage forms a C-terminus in S1 protein with a ^682^RRAR^685^ that can interact with RXXROH (where R is arginine and X is any amino acid; R may be substituted by lysine). This terminus sequence conforms to the C-end rule (CendR) and is a ligand for neuropilin receptors 1 and 2 (NRP1 and NRP2). Cryo-electron microscopy demonstrated that S1/S2 is a constituent of a loop that may be bound to receptors [50,51]. Extracellular regions of NRP1 and NRP2 contains the b1 do-main which has the specific binding site for CendR peptides. After cleavage action, the virus fuses to the cell membrane and enters into the cytoplasm via endosomic transport. Valid SARS-2-S activation by TMPRSS2 and airway epithelium cell entry are related to S1/S2 priming [52]. Eighteen proteases, many of which are coded in human airways, are part of the type II transmembrane serine protease (TTSP) family, in which TMPRSS2 is included. Two recent analyses showed that TMPRSS13 is a second prominent activator of the SARS-CoV-2 S protein [53,54]. Abbreviations: ACE-2: angiotensin-converting enzyme 2; CAT B/L: catalase B/L; NRP 1: neuropilin-1; NRP 2: neuropilin-2; RRAR Arg–Arg–Ala–Arg sequence; RXXR: where R is arginine and X is any amino acid (R may be substituted by lysine); S1: spike glycoprotein 1; S2: spike glycoprotein 2; TMPRSS-2: transmembrane serine protease 2.

Transmission of SARS-CoV-2 occurs via inhalation of respiratory microdroplets from individuals infected with SARS-CoV-2. Once in the host, SARS-CoV-2 enters the cell using methods common to other viruses [46]. The spike protein (S) binds the virus to the ACE2 receptor on the surface of the cell [47] (Figure 1).

TMPRSS2 is fundamental for viral entry into target cells and spread in the infected host, but an additional system for cell entry is the S protein which can use the endosomal cysteine proteases CatB/L. Hofmann et al. [52] demonstrated that TMPRSS2 activity was only inhibited, but not eliminated using camostat mesylate, reflecting a residual S protein priming by CatB/L.

A special concern is related to temperature influence on viral replication. This can be effective throughout the airway tract ranging from 30–32 °C in the nose to 37 °C in the deeper airways. Considering the abundant replication of SARS-CoV-2 in the nose, it may be assumed that S protein is fine-tuned in this anatomical region. Many studies investigated this feature dedicating their observations to spike protein mutations [55,56,57,58]. Mutation S^G614^ became predominant within four months of the beginning of the pandemic. A high-level load in the upper airways is suggestive of more transmissibility. This has been ascribed to protein stability, increased level of the open spike conformation, and a more efficient proteolytic activation of the S protein. Two particular mutations of spike proteins are related to variants Asp (D) or Gly (G) at residue 614. For both strains, infectivity remained largely stable at 33 °C. At 37 °C, both viruses deteriorated, but the decline was faster for the S^D614^ strain. It is worth noting that, at 37 °C, its infectivity was 10-fold (day 3 p.i.) to 35-fold (day 4 p.i.) lower than at 33 °C (*p* < 0.0001), while the S^G614^ virus was 2.7–7-fold less infectious at 37 °C versus 33 °C (*p* = 0.02). This indicates that mutation S^G614^ has a key role in the stability of S protein at 37 °C [59].

The pH may have a specific role in spike stability [59]. The lumen of the bronchi (pH ~ 7.5) is less acidic than the nasal cavity (pH ~ 6.3). The latter pH (6.3) has a more stabilizing effect than the former (pH 7.5). This was tested for almost all pseudoviruses. Between pH 7.5 and 8.0, no significant difference in spike stability was observed. One exception concerned the test on SARS-S, which had the highest stability at pH 7.5 and a lower infectivity at pH 6.3 (*p* = 0.0014). A similar pH influence was noted in the two variants of SARS-2-S.

### 3.2. Host Response: How NETs Interfere

The host may exhibit distinctive clinical features of severe COVID-19 following SARS-CoV-2 infection. Clinically, we mainly recognize acute respiratory distress syndrome, neutrophilia, and the cytokine storm, along with severe inflammatory response syndrome or sepsis with multi-organ involvement. The extreme inflammatory response elicited in the host by SARS-CoV-2 has recently aroused great interest, with particular emphasis on the excessive activation of NETs, cytokine storm, and sepsis. Multi-organ damage is caused by the combination of these three factors.

The role of neutrophils in COVID-19 disease severity has been well studied. Evidence suggests that neutrophil activators such as IL-8 and G-CSF and effectors including resistin, lipocalin-2, and hepatocyte growth factor are early expressed biomarkers in patients with the severe form of COVID-19. Furthermore, a substantial link in the relationship between high levels of immature granulocytes/neutrophils and increased mortality [60] was disclosed.

SARS-CoV-2 can cause the release of neutrophil extracellular traps (NETs) by neutrophils [61]. In a landmark paper, Brinkmann anticipated the role of NETs [62], which embody not only chromatin fibers but also enzymes such as neutrophil elastase, cathepsin G, and myeloperoxidase [63,64]. NETs represent an outpost against infections with the specific action of immobilizing and degrading bacteria, fungi, viruses, being a critical effector mechanism for containing infections [65]. However, the nonunique role of NET in immunity has been revealed, with a dual effect, pro- or anti-inflammatory [66,67]. Aggregates of NETs reduce inflammation, leading to the degradation of cytokines and chemokines [68]. Regarding the tissue damage due to NETs, it was revealed during infection with *Escherichia coli* that an interaction between NETs and platelets caused tissue damage [69]. Patients with COVID-19 experience a high level of NETs in plasma [22,70,71], correlated with a greater severity of the disease [71], evidenced by the occurrence of critical lung damage and microvascular thrombosis [70].

Concern about vascular occlusion caused by NETs involves several target tissues: lung [72], kidney, liver [73], and heart. This suggests that the thrombotic effects of NETs could be responsible for the systemic and harmful effects present in critically ill patients with COVID-19. A synergistic role with NET was also evoked by the activation of the complement system. In patients with COVID-19, it has been disclosed that inhibition of C3 [74] and C5 [75] reduced NET release. Marked coagulation dysregulation is the cause of a worse prognosis in COVID-19 [76,77,78], and both NETs and complement proteins are associated with these thrombotic events [75]. A new frontier in COVID-19 therapy stems from research on the triple complement–NET coagulation interaction.

**Figure 2:**
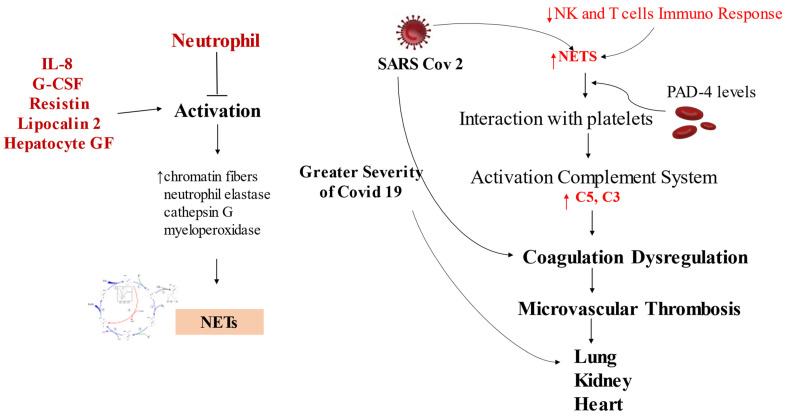
SARS-CoV-2 causes activation of neutrophils mediated by IL-8, G-CSF, resistin, lipocalin-2, hepatocyte growth factor and NET release. The immune response of NK and T lymphocytes con-tributes to the formation of NETs with the activation of the complement system (C5 and C3). The result is the development of microvascular thrombosis, which leads to organ damage. Abbreviations: C, complement; GF, growth factor; IL, interleukin; NK, natural killer cells. Other abbreviations are provided in the text. Arrows explain the increase or decrease of relative component.

Genetics has taught us that abundant NET formation in patients with COVID-19 is sustained by higher transcriptional level [79]. Investigators hypothesized that the transcriptional increase assets may be related to a negative regulatory mechanism of the host’s immune response of natural killer cells (NK) and T cells, with a consequential reduction in the antiviral response [52]. The main cause of this altered response results in the clinically more severe forms of COVID-19, in which both circulating and lung neutrophils have been found to release high levels of NET. There is evidence that this phenomenon is exacerbated by a direct action induced by SARS-CoV-2 in favoring the release of NETs [71]. Furthermore, this NET release is linked to PAD-4 levels [71]. PAD4 plays a pivotal role in the constitution of NETs, which is due to the hypercitrulination process of histones, with consequent decondensation of chromatin caused by PAD [80] (Figure 2).

The spectrum of work of neutrophils activated by SARS-CoV-2 is broad since they can induce apoptosis of A549 cells of the pulmonary epithelium and myocardial tissue [15,23], thus strengthening the role played by neutrophils in COVID-19 immunopathology and other infections from coronavirus [71].

## 4. COVID Infection and Cardiovascular Implications

**Figure 3:**
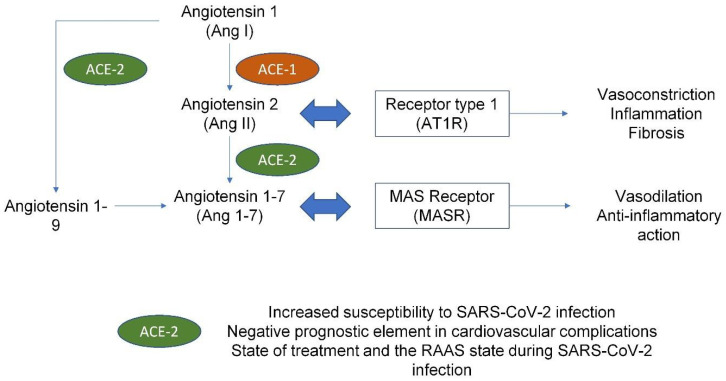
Renin–angiotensin–aldosterone system: summary of pathophysiologic effects and its importance in SARS-CoV-2 infection. Abbreviations: ACE: angiotensin-converting enzyme, type 1 or type 2; AT1R: angiotensin receptor type 1; MASR mitochondrial assembly receptor; RAAS: renin–angiotensin–aldosterone system; SARS-CoV-2: severe acute respiratory syndrome coronavirus 2.

Clinical evidence underlines that those with cardiovascular diseases are at risk or have a more severe illness due to SARS-CoV-2 infection than the general population [81]. Patients with coronary artery disease (CAD) or impaired left-ventricular function have increased risk of developing major cardiac injury, requiring hospitalization or intensive treatments, as they have pre-existing alterations of the renin–angiotensin–aldosterone system with upregulation of ACE receptors. The increased number of ACE receptors upon the surface cell makes them more prone to virus entry as this receptor is used as a gateway [82,83] (Figure 3).

In this context, exogenous ACE-2 activation limits thrombus formation and platelet aggregation, as well as attachment to vessels [84,85]. Elevated values of ACE-2 are related to an increased susceptibility to SARS-CoV-2 infection and are generally considered a COVID-19-specific negative prognostic factor [86,87]. Plasma ACE-2 and angiotensin peptides levels may also indicate the progress of treatment and the RAAS state during COVID-19. Earlier studies established that a soluble form of recombinant human ACE-2 (rhACE-2; APN01 (0.4 mg/kg, IV, BID for 7 days), GSK2586881: 0.4 mg/kg, IV, BID for 3 days) neutralized excessive SARS-CoV virus and enhanced the protective cellular action of ACE-2 in ARDS patients [88,89]. ACE inhibitors (ACEi) upregulate ACE-2 expression on the cell surface, and this may improve the survival rate in COVID-19 patients [83], maintaining Ang II degradation, which can decrease AT1R activation.

Myocardial injury is a major contributor of mortality in COVID. In a study per-formed in hospitals in Wuhan, China, a high percentage mortality (70%) was reported in patients with high cTnI levels. Acute inflammation stimulus triggered by SARS-CoV-2 infection is embedded in atherosclerotic plaque development and progression [90]. This problem in SARS-CoV-2 is directly related to an acute inflammatory stimulus, triggered by virus infection. Development and destabilization of atherosclerotic plaque may induce acute myocardial infarction (AMI). These data are confirmed by many studies, particularly those performed in China [91,92,93,94]. A particular role in ischemic heart disease is represented by the so-called “cytokine storm” [95]. Proinflammatory cytokines elicited from endothelial cells cause a change in homeostatic functions and may result in endothelial impairment, subsequent destabilization of the atherosclerotic plaque, and thrombosis. Cytokines such as IL-1α, IL-1β, IL-6, and TNF-α can perturb all of the protective functions of the normal endothelium and potentiate the pathological processes.

**Figure 4:**
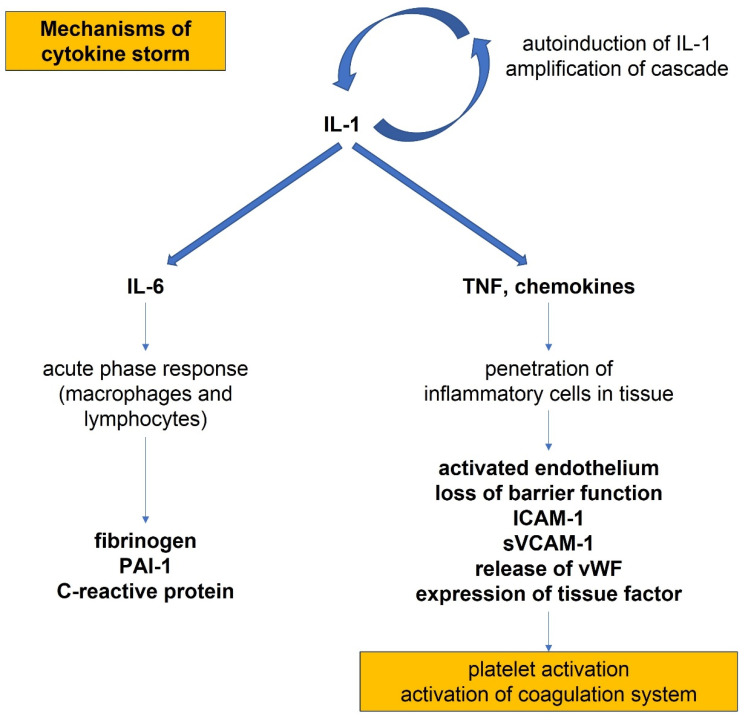
Mechanisms of cytokine cascade and activation of the coagulation system in SARS-CoV-2 infection, showing the autoinduction of interleukins and the amplification of the inflammatory cascade. Abbreviations: IL: interleukin; PAI: plasminogen activator inhibitor; TNF: tumor necrosis factor alpha; ICAM: intercellular adhesion molecule; sVCAM: serum vascular cell adhesion molecule; vWF: von Willebrand factor.

The pathophysiological mechanism of a cytokine storm is centered on the autoinduction of proinflammatory cytokine IL-1 (Figure 4).

IL-1 can induce its own gene expression, precipitating an amplification that leads to a cytokine storm [96,97,98]. IL-1 induces also the expression of other proinflammatory cytokines including TNF-α. The invasion of IL-1 and leucocytes can elicit the production of chemoattractant molecules including chemokines that provoke the penetration of inflammatory cells into tissues [99]. In the meantime, IL-1 stimulates the production of IL-6. IL-6 is a 27 kDa cytokine involved in a variety of immune and inflammatory responses. Plasma levels are generally very low. During acute infection, a large variety of cells including macrophages, as well as B and T lymphocytes, increase the production of IL-6. In addition to local effects, IL-6 provides a proximal stimulus to the acute phase response. IL-6 induces the synthesis of fibrinogen, the precursor of clots, PAI-1, a major inhibitor of the endogenous fibrinolytic mediators, and C-reactive protein, an inflammation biomarker strictly linked to COVID-19 [100]. During infection, the endothelium becomes activated, resulting in a loss of barrier function, expression of adhesion molecules such as soluble ICAM-1 (intercellular adhesion molecule 1) and soluble VCAM-1 (vascular cell adhesion molecule 1), release of VWF that allows binding of platelets, and expression of TF that activates the coagulation system.

## 5. Dysregulation of Hemostasis Induced by SARS-CoV-2

In individuals experiencing severe forms of COVID-19, abnormal blood clots can form due to hemostasis disorders, ranging from pulmonary embolisms in the lungs and deep vein thrombosis in the legs to the formation of clots leading to strokes or heart at-tacks. High plasma D-dimer level is deemed to be an independent risk factor for death [91,101,102,103]. Substantial evidence has shown that vascular complications are more frequent in individuals with cardiovascular comorbidity and with autoimmune diseases [11,12,16,32,33,35]. Although disseminated intravascular coagulation has been identified as the primary disorder in COVID-19 coagulopathy, the majority of patients preserved normal concentrations of coagulation factors, fibrinogen, and platelets. Therefore, investigators suggested that COVID-19 drives a distinctive prothrombotic state that is unlike conventional representations of sepsis-induced coagulopathy [104,105]. The hemostatic disorder presentation is variable, with the development of arterial thrombosis including strokes and myocardial infarctions [106,107]. Histopathological examination of lung specimens from patients with severe disease revealed not only the characteristic signs of acute respiratory distress syndrome (ARDS) but also evidence of occlusion of small blood vessels due to accumulation of fibrin [108,109,110]. The possible synergistic mechanisms via which SARS-CoV-2 infection in patients with severe acute respiratory syndrome can cause macrovascular and microvascular thrombosis are manifold [111]. Considerable work in inducing the dysregulatory mechanism of coagulation is due to the cytokine storm that activates leukocytes, endothelium, and platelets. In support of cytokines, hypoxic vaso-occlusion and direct activation of immune and vascular cells by the viral infection also occur. In addition, NETs in the blood have been noted in many patients with COVID-19 admitted to hospital with critical illness [22,112]. NETs as remnants of inflammatory cells can also contribute to the prothrombotic environment [14,70,73,113].

### 5.1. Hypercoagulability in COVID Patients

Coronary thrombosis may be ascribed by the hypercoagulable state and endothelium impairment in COVID-19. Several studies reported coagulation abnormalities: elevation of fibrinogen-derived peptides (FDP) such as D-dimer, slight elevation of aPTT, and elevated levels of von Willebrand factor (vWF), fibrinogen, and factor VIII [114,115,116]. Furthermore, immunohistochemical investigations confirmed an overexpression of the FVIII [117,118]. These humoral characteristics lead to extrinsic coagulation cascade activation and, to a lesser effect, activation of the intrinsic cascade [114,115,116].

During viral infection, endothelium impairment may cause alterations of physiological balance and shift normal function toward vasoconstriction, inflammation, and thrombosis [119]. Normally, the endothelial layer has quiescent endothelial cells, which express a low level of tissue factor activity, the primary stimulant of the extrinsic coagulation cascade, resulting in anti-inflammatory and anticoagulant action [120,121].

Anti-inflammatory action is also obtained by endothelial cell inhibition of their interaction with immune cells and platelets, in addition to producing coagulation inhibitors and fibrinolytic enzymes. Anticoagulation occurs through endothelial cell surface expression of a glycocalyx and glycolipid matrix [122].

After endothelial cell damage, TF is overexpressed, factor VII is converted to VIIa, and fibrin is formed as a consequence of this cascade. Recent evidence implicates endothelial damage (endotheliopathy) in patients with severe COVID-19 [123,124].

The quoted cytokine storm is crucial in this process since cytokines are strictly connected to the activation of TF. SARS-CoV-2, especially in patients admitted to intensive care units (ICUs), induces increasing serum levels of IL-1, IL-2, IL-6, IL-7, IL-10, TNFα, macrophage colony-stimulating factor (M-CSF), granulocyte colony-stimulating factor (G-CSF), granulocyte-macrophage colony-stimulating factor (GM-CSF), interferon-gamma-induced protein (IP-10), monocyte chemoattractant protein-1 (MCP-1), and macrophage inflammatory protein 1 (MIP 1) [125,126,127,128]. After cytokine production, an increase in TF mRNA is detected inside the endothelial cells, in addition to an increase in TF surface expression. Moreover, TF-containing microparticles are developed by the cells. TNF-alpha has a particular role in elevating TF mRNA through the TNF receptor. In addition to these mechanisms, SARS-CoV-2 may induce tissue hypoxia, ischemic tissue injury, and secondary increment of TF from endothelial cells and other cells including monocytes, neutrophils, and platelets [120,129,130]. An autopsy examination of lungs of COVID-19 patients using scanning electron microscopy demonstrated severe endothelial injury caused by the presence of intracellular virus and alteration of cell membranes [131]. Goshua et al. reported a single-center study that strengthened the idea of endothelial cell damage by viral invasion. These authors underlined the correlation between blood elevation of markers of endothelial cell damage such as VWF antigen and thrombomodulin and markers of platelet activation such as soluble *p*-selectin derived from Weibel–Palade bodies in 68 patients with COVID-19. In this series, 48 patients were in the ICU and 20 were non-ICU patients. These data were compared to 13 non-hospitalized and asymptomatic controls. The markers of endothelial cell and platelet activation were significantly elevated in ICU patients in comparison with non-ICU patients. Mortality was significantly higher in patients with elevated VWF antigen and soluble thrombomodulin [114].

Elevated plasma VWF concentrations are suggestive of high endothelial cell activation and indicate strong platelet activation [123].

In COVID-19 patients, the activation of intravascular coagulation and fibrinolytic processes is similar to that seen in disseminated intravascular coagulation (DIC), a severe pathological condition related to consumptive coagulopathy [132].

**Figure 5:**
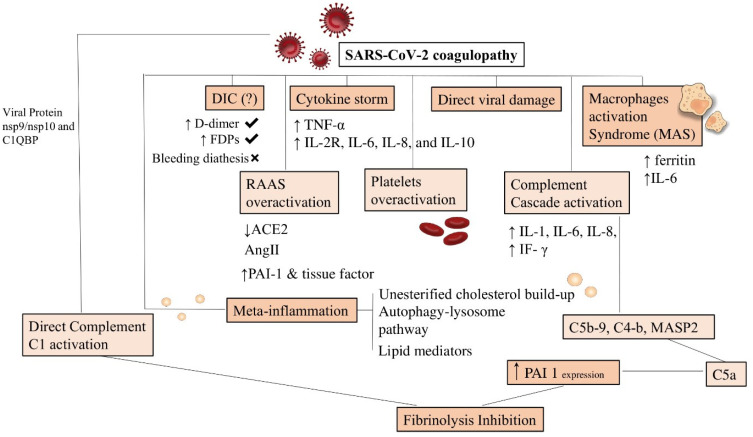
Summary of the dysregulations in coagulation system after SARS-CoV-2. SARS-CoV-2 coagulopathy is supported by the following pathological events: DIC, cytokine storm process, and direct action of the virus, inducing damage and activation of macrophages. RAAS overactivation associated with platelet and complement overactivation (direct and indirect) leads to fibrinolysis inhibition. Abbreviations are as shown in previous figures. Arrows explain the increase or decrease of relative component.

Hypercoagulability in SARS-CoV-2 patients should also be referred to as activated complement cascade [133,134]. Vascular deposits of C5b-9 (membrane attack complex), C4-b, and mannose-binding protein-associated serine protease 2 (MASP2) are markers of complement activation [135]. C5-a can directly provoke the extrinsic coagulation cascade and can inhibit the fibrinolysis process by upregulating PAI-1 expression. Li et al. demonstrated an interaction between viral protein nsp9/nsp10 and C1Q-binding protein (C1QBP) [136]. This event induces the activation of complement C1 (Figure 5).

Platelets carry out crucial work in thrombosis. In patients with COVID-19, a higher platelet volume is recorded, and platelets tend to bind more fibrinogen than in normal hemostasis [137]. In humans, less than 55% of unstimulated platelets have p-selectin positivity. In COVID-19 patients, nonactivated platelets have higher levels of p-selectin, CD-63, and TF expression, as well as and thrombin receptor-activating peptide (TRAP) [138,139]. During SARS-CoV-2 infection, platelets show a higher level of aggregability and develop greater interactions with neutrophils, monocytes, and T cells. In addition, upregulation of mitogen-associated protein kinases (MAPKs) and increased generation of thromboxane are also noted [140].

### 5.2. The Role of NETs in Coronary Thrombus Formation

Monocytes and neutrophils are involved in thrombosis related to SARS-CoV-2 infection. Nuclear chromatin, nuclear histones, and granular antimicrobial proteins contribute to the NET [141]. NETs may contribute to TF and sC5-b9 expression, contributing to the coagulation cascade and thrombus formation. Moreover, NETs may express TF on their surface [75,142]. NET formation in COVID-19 has a particular role in the occlusion of pulmonary microvessels and, consequently, organ or tissue damage. The neutrophil subset prone to producing NETs is represented by low-density granulocytes (LDG), a very important factor in the predisposing proinflammatory and procoagulant state, although involvement of normal density granulocytes (NDG) is also noted in NET formation. Enhanced formation and low clearance of NET have a central role in tissue damage during infectious diseases. Circulatory NET complexes are related to microthrombus development [70]. The percentage of mature and immature LDG is higher in patients who are critically unwell. Immature LDG expresses a phenotype without CD10. A strict correlation has been noted between LDG and serum expression of TGF beta-2, VEGF, TNF-alfa, IL-8, IL-15, and IL-18. Circulating NETs in COVID-19 sera contain LL-37-DNA and high-mobility group box 1 (HMGB1)-DNA. Instead, a low plasma level of interferon-stimulated gene 15 (ISG-15) DNA is registered. The lack of plasma degradation of NETs is relevant in SARS-CoV-2 infection as it is more pronounced in the severe form of infection. Autoimmunity has been advocated during COVID-19. During infection, a high level of anti-NET IgG was discovered, and this feature was linked to ANCA and ANA formation. LDG and NDG NETs induce proinflammatory activation in monocyte-derived macrophages. In fact, in almost all patients with a severe form of infection, a large number of cytokines are released by this mechanism [143].

**Figure 6:**
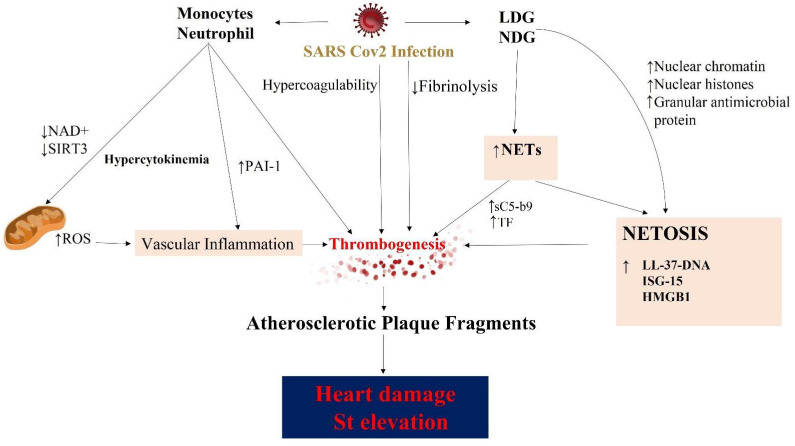
Summary of the mechanism that induces heart damage from NET formation in patients with severe COVID-19. Three distinct processes result in heart damage: vascular inflammation, thrombogenesis, and NETosis. Abbreviations: HMGB1, high-mobility group box 1; ISG-15; interferon-stimulated gene; LDG, low-density granulocyte; LL37, active cathelicidin; NDG, normal density granulocyte; NAD, nicotine adenine dinucleotide; ROS, reactive oxygen species; SIRT3, Sirtuin 3. Other abbreviations are as shown in previous figures. Arrows explain the increase or decrease of relative component.

In the above-quoted study of Blasco et al. [23], all COVID-19 patients had a burden of NETs in thrombi, and this was significantly higher in those with SARS-CoV-2 infection than infection-free patients with STEMI. It is worth noting the immuno-histochemical analysis which elucidated the absence of atherosclerotic plaque fragments in thrombi from patients with STEMI and COVID-19. These were composed of polymorphonuclear cells and fibrin, unlike most coronary thrombi from STEMI patients without infection. In the mentioned study, plasma alteration was signified by prolongation of prothrombin time, high D-dimer concentration, and undetectable platelet counts. This may strengthen the role of neutrophils and NET in thrombus formation and progression. A link between NET and unfavorable outcomes has also been suggested [23,144] (Figure 6).

### 5.3. Antiphospholipid Syndrome and NETs: A Growing Concern for Microcirculation

One in 2000 people suffer from antiphospholipid antibody syndrome, which is a common form of acquired thrombophilia [145]. Patients who experience this disease have durable autoantibodies against phospholipids and phospholipid-binding proteins (aPL antibodies), such as prothrombin and β2 I glycoprotein (β2GPI). The action of these antibodies is mainly directed toward cell surfaces, inducing the activation of endothelial cells, platelets, and neutrophils [146,147]. Given this functional reversal, the blood and endothelium become, like an innocent bystander, the interface toward thrombosis. The peculiar characteristic of producing autoantibodies gives antiphospholipid syndrome the potential to direct the thrombotic process in vascular districts of different sizes, thus involving ramifications in the arterial and venous tissue. The most severe form of antiphospholipid antibody syndrome often has fatal consequences and has revealed similarities to the diffuse coagulopathy recorded in patients with the severe form of COVID-19 [148].

Since 2006, new classification criteria have been available for antiphospholipid syn-drome, diagnosable through the detection of persistently positive anti-cardiolipin (aCL antibodies) or anti-β2GPI (aβ2GPI antibodies) autoantibodies [149]. To complete the diagnosis of thrombophilia in COVID-19 patients with coagulopathy, the lupus anticoagulant test is also used among the diagnostic criteria. This functional test selects aPL antibodies on the basis of their anomalous potential to extend in vitro clotting tests and records the presence of heterogeneity between aPL antibody species including anti-phosphatidyl-serine/prothrombin autoantibodies (aPS/PT antibodies) [150].

Several case reports and case series have suggested a correlation between the formation of aPL antibodies in patients with COVID-19 and their possible relationship with thrombosis [151,152,153,154,155,156]. The evidence revealed that viral infections cause transient production of aPL antibodies [157,158,159,160]. However, in the COVID-19 era, measuring the pathogenicity of these short-lived autoantibodies as an additional factor in aggravating the disease has become a fundamental line of investigation.

Zuo and colleagues [161] recorded various antiphospholipid (aPL) antibody types in serum samples from 172 patients admitted to hospital with severe COVID-19. Investigators revealed the antibodies in 52% of samples using the manufacturer’s threshold and in 30% of samples when they used a stricter cutoff (≥40 ELISA-specific units). In addition, in the serum samples, they recorded rates of 24% aPS/PT IgG, 23% anticardiolipin IgM, and 18% aPS/PT IgM.

Higher aPL antibody levels were associated with severe respiratory disease, poorer kidney function, myocardial injury, and hyperactivity of the immune system, including the release of NETs. It should be noted that the authors previously disclosed that NETs were increased in patients with COVID-19.

We learned that neutrophils release NETs to counteract infections, but the traps can encourage uncontrolled inflammatory response and clot formation in cases of pathologic dysregulation. Clearly, this incipient evidence on NETs and autoantibody constitution amplifies the yield of action against COVID-19 [161,162]. The points outlined below deserve consideration.

First, several studies showed that neutrophils from healthy people overproduced NETs when cultured with autoantibodies from patients with severe acute respiratory syndrome due to SARS-CoV-2 infection [162,163,164]. Second, a similar response was found with aPL antibodies from patients with APS [165]. Findings proving that COVID-19 triggers the production of autoantibodies that ultimately lead to thrombosis have also been published. Shi and colleagues reported that injections with antibody fractions from patients with severe COVID-19 drove more aggressive thrombosis [166].

Whether these antibodies provide a therapeutic target or can be used to further clarify the extent of vascular damage and associated morbidity or mortality remains to be investigated in depth. Detecting these antibodies in patients with COVID-19 can also help identify a population at greater risk of developing severe forms of thrombosis, leading to complications such as stroke and myocardial infarction and, thus, being able to benefit from aggressive anticoagulation therapy during the clinical and surgical management of COVID-19 patients [23,92,167,168,169,170,171,172].

Interfering with the blockade of NETs that are released in response to these autoantibodies could potentially be useful in preventing the cascade of events responsible for the production of clots in patients with COVID-19 in whom myocardial infarction occurred [23]. The primary therapeutic strategies aimed at controlling hyperinflammation with the use of steroids, such as dexamethasone and nonsteroidal anti-inflammatory drugs (NSAIDs) have been administered in a series of clinical trials. As regards the administration of NSAIDs, aspirin for its antiplatelet action can be significantly associated with a reduced risk of NET release during COVID-19 and the thrombotic complications associated with combining anti-inflammatory and anticoagulant action [173]. It is therefore possible to speculate on a substantial role of aspirin in reducing NET formation and related immunothrombotic events.

Recently, a study revealed that dipyridamole blocks NET release in mice, paving the way for the potential use of this drug as a treatment for APS. The authors found that dipyridamole interferes by reducing the release of NETs from neutrophils exposed to COVID-19 autoantibodies [174]. Promising results derived from a Chinese study suggest that dipyridamole suppresses SARS-CoV-2 replication [175] in vitro. In 31 enrolled patients who received dipyridamole, a significantly decreased concentration of D-dimers (*p* < 0.05), increased lymphocyte and platelet recovery in the circulation, and markedly improved clinical outcomes were noted vs. the control patients [175].

This recent promising evidence on the immunomodulatory and potentially antiviral properties of dipyridamole, mediated by a robust antiviral type I interferon immune response [174] and supported by the fact that dipyridamole is a safe and inexpensive antiplatelet medication, prompted the DICER (Dipyridamole to Prevent Coronavirus Exacerbation of Respiratory Status) RCT aimed at demonstrating the effectiveness and safety of dipyridamole among patients hospitalized with COVID-19 (ClinicalTrials.gov Identifier: NCT04391179) [176].

### 5.4. The Role of Lipoprotein (a) and IL 6 in Arterial Thrombosis

Lipoprotein (a) (Lp (a)) is composed of an LDL particle bound to apolipoprotein (a) (Apo (a)). Lp (a) is an independent risk factor for CVD. Plasma values of Lp (a) are genetically determined; environmental factors have a minor role in the expression of this lipoprotein.

Apo (a) is highly homologous to plasminogen but lacks fibrinolytic activity. Activation of plasminogen to plasmin is inhibited by the Apo (a) structure of Lp (a). Clot lysis is also altered by Apo (a) competition for binding loci to fibrin by plasminogen and plasmin. Increasing PAI-1 and inactivation of tissue factor pathway inhibitor (TFPI) are added prothrombotic features of Lp (a). TPFI inactivation provokes factor VII expression with enhanced blood coagulation. [177,178].

The aforementioned risk factors for cardiovascular disease may be more prevalent in patients who develop SARS-CoV-2 infection. There is an extensive relationship linking Lp (a), systemic inflammation, and proatherogenic and prothrombotic status.

IL-6 is involved in host defense action through acute-phase proteins and immunoglobulins [179], and systemic activation is sustained by an IL-6 response element (RE) CTGGGA in many genes such as LPA [180]. The LPA gene includes the promoter of five IL-6 REs, but only IL-6 RE6 upregulates Apo (a) synthesis [181]. During COVID-19 infection, IL-6 plasma levels are 20-fold higher than baseline levels. Hepatic Apo (a) synthesis and subsequent Lp (a) secretion in blood are induced by a high plasma level of cytokines [182].

Many clinical trials evaluated the association of IL-6 concentration with Lp (a). Horvath et al. [183] described a strong link between Lp (a) and plasma IL-6. Additional clinical evidence demonstrated that patients with high levels of IL-6 were more prone to a high plasma level of Lp (a). This feature was particularly highlighted during the approval of IL-6 receptor (IL-6R) monoclonal antibody (mAb) tocilizumab [184,185,186]. The link between IL-6 response genes and LPA gene expression in vivo is evident from transcriptomic analysis of human liver biopsies.

## 6. Conclusions

COVID-19 infections affect the cardiovascular system in several ways. The structure and anatomy of the virus are pivotal for its virulence in comparison to other *Coronaviridae*. In particular, the host interaction and response may explain the variability of severity in patients. Angiotensin-converting enzyme 2 (ACE-2) activation may be implicated in the cardiovascular and thrombogenic potential of the disease. The virus may also have direct effects on the endothelial lining, affecting hemostasis and resulting in thrombosis through several mechanisms. Three distinct processes result in heart damage: vascular inflammation, thrombogenesis, and NETosis. The role of the ACE2 pathway, NET generation, and ongoing thrombosis should be identified as therapeutic targets to reduce the severity of disease. These agents, including dipyridamole, may be used in routine practice following the conclusion of such trials.

## Figures and Tables

**Table 1:** SARS CoV-2 strains. G and GR variants are predominant in Europe, while GH is more common in North America.

SARS-CoV-2 Strains	Description
L strain and the similar ORF8-L84S Strain	originated in Wuhan, China
S strain	mutation of ORF8, L84S
V strain	variant of ORF3a-coding protein NS3, G251V
G strain	mutation in spike protein, D614G
GH strain	mutations in spike protein, D614G and ORF3a, Q57H
GR strain	mutation in nucleocapsid gene, RG203KR

## Data Availability

Not applicable.

## Abbreviations/Acronyms

Apo (a)	apolipoprotein (a)
ACE1	angiotensin-converting enzyme 1
ACE2	angiotensin-converting enzyme 2
ACEi ACE	inhibitors
aCL	anticardiolipin
ANA	antinuclear antibodies
ANCA	antineutrophil cytoplasmic antibodies
aPL	antiphospholipid
aPS/PT Ab	anti-phosphatidylserine/prothrombin autoantibodies
APS	antiphospholipid syndrome
ARDS	acute respiratory distress syndrome
AT1R	angiotensin type 1 receptor C1QBP
C1Q	binding protein
CatB/L	cysteine proteases cathepsin B/L
CendR	C-end rule
COVID-19	Coronavirus disease 2019
DIC	disseminated intravascular coagulation
DICER	Dipyridamole to Prevent Coronavirus Exacerbation of Respiratory Status
E	envelope
FDP	fibrinogen-derived peptide
G-CSF	granulocyte colony-stimulating factor
GM-CSF	granulocyte-macrophage colony-stimulating factor
ICAM-1	intercellular adhesion molecule 1
ICU	intensive care unit
IL	interleukin
IP-10	interferon-gamma-induced protein
ISG-15	interferon-stimulated gene 15
LDG	low-density granulocytes
LDL	low-density lipoprotein
Lp(a)	lipoprotein a
M	membrane protein
mAb	monoclonal antibody
MAM	domain in meprin, A5, receptor protein tyrosine phosphatase mu
MAPK	mitogen-associated protein kinase
MASP2	mannose-binding protein-associated serine protease 2
MAS-R	mitochondrial assembly receptor
MCP-1	monocyte chemoattractant protein 1
M-CSF	macrophage colony-stimulating factor
MERS-CoV	Middle East respiratory syndrome coronavirus
MIP 1	macrophage inflammatory protein 1
N	nucleoprotein
NDG	normal-density granulocytes
NET	neutrophil extracellular trap
NRP1	neuropilin receptor 1
NRP2	neuropilin receptor 2
Nsps	nonstructural proteins
ORF	open reading frame
PAD	peptidyl arginine deaminase
PAI	platelet activator inhibitor
Pp	polyprotein
RAAS	renin–angiotensin–aldosterone system
RE	response element
rhACE2	recombinant human
ACE2 S	spike
SARS-CoV-2	severe acute respiratory syndrome coronavirus 2
STEMI	ST-elevation myocardial infarction
TF	tissue factor
TFPI	tissue factor pathway inhibitor
TGF-β2	transforming grow factor beta 2
TMPRSS2	transmembrane serine protease 2
TNF	tumor necrosis factor
TRAP	thrombin receptor-activating peptide
TTSP	transmembrane serine protease
UTR	untranslated region
VCAM-1	vascular cell adhesion molecule 1
VWF	von Willebrand factor
β2GPI	β2 I glycoprotein
